# Quantifying Laryngopharyngeal Reflux in Singers: Perceptual and Objective Findings

**DOI:** 10.1155/2017/3918214

**Published:** 2017-09-19

**Authors:** Adam T. Lloyd, Bari Hoffman Ruddy, Erin Silverman, Vicki M. Lewis, Jeffrey J. Lehman

**Affiliations:** ^1^University of Miami, Miller School of Medicine, 1121 NW 14th Street, 3rd Floor, Miami, FL 33136, USA; ^2^Ear, Nose, Throat and Plastic Surgery Associates, University of Central Florida, HPA II-101, 4364 Scorpius Drive, Orlando, FL 32816-2215, USA; ^3^Division of Pulmonary, Critical Care, and Sleep Medicine, College of Medicine, University of Florida, P.O. Box 100225, 1600 SW Archer Rd., Gainesville, FL 32610-0225, USA; ^4^Ear, Nose, Throat, and Plastic Surgery Associates, 44 W. Michigan St., Orlando, FL 32806, USA

## Abstract

This study examines the relationship between laryngopharyngeal reflux (LPR) symptoms and oropharyngeal pH levels in singers. We hypothesized that reported symptoms would correlate with objective measures of pH levels from the oropharynx, including the number and total duration of reflux episodes. Twenty professional/semiprofessional singers completed the Reflux Symptom Index (RSI) and underwent oropharyngeal pH monitoring. Mild, moderate, or severe pH exposure was recorded during oropharyngeal pH monitoring. Correlations were performed to examine potential relationships between reflux symptoms and duration of LPR episodes. Symptom severity did not correlate with pH levels; however, we found a number of covariances of interest. Large sample sizes are necessary to determine if true correlations exist. Our results suggest that singers may exhibit enhanced sensitivity to LPR and may therefore manifest symptoms, even in response to subtle changes in pH. This study emphasizes the importance of sensitive and objective measures of reflux severity as well as consideration of the cumulative time of reflux exposure in addition to the number of reflux episodes.

## 1. Introduction

Laryngopharyngeal reflux (LPR) refers to retrograde movement of gastric contents into the larynx, pharynx, and upper aerodigestive tract [1] and is commonly associated with a number of voice disorders, particularly among singers [2–4]. Common symptoms of LPR include hoarseness, throat clearing, the perception of excessive mucous accumulation within the throat, difficulty swallowing, breathing difficulties, globus sensation, cough, persistent “tickle” sensation within the throat, sore throat, and regurgitation [1, 5]. Less common upper airway symptoms include worsening asthma, wheezing, shortness of breath, dental hypersensitivity, laryngospasm, nausea, otalgia, muscle spasms, bronchospasm from aspiration, and halitosis [6].

Singing requires a high magnitude of recruitment and activation of respiratory and laryngeal structures. Tasks which emphasize coordinated contractions of the diaphragm and intercostal and abdominal muscles may place singers at an elevated risk for developing LPR due to high-magnitude changes in intrathoracic pressures that may occur during such maneuvers. During inspiration, the thoracic cavity expands and the diaphragm compresses the stomach, putting pressure against the LES, potentially causing stomach acids to reflux into the esophagus. There is a similar effect during prolonged expiration, as with singing, as the abdominal muscles are activated and exert pressure against the stomach wall as the thoracic cavity compresses. These pressures can affect lower esophageal sphincter opening and closing (LES), potentially contributing to LES dysfunction [2–4]. Consequently, individuals who engage in singing as a primary professional activity, frequently display higher reflux symptom scores [2, 3, 7, 8]. In addition to the actions of the LES, a wide range of other physiological processes relating to gastrointestinal function may be affected, potentially resulting in hyperacidity and esophageal dysmotility [6]. Performance-related stress and anxiety exert a disproportionate effect on singers [9–13]. Additionally, external influences such as irregular eating habits (e.g., eating late at night or following rehearsals or performances), or inconsistent sleep schedules, may further exacerbate these underlying vulnerabilities, potentially placing singers at increased risk for LPR.

Antireflux medications are typically the first line of treatment for singers who report symptoms consistent with LPR [4, 10, 14]. Typical antireflux medications include over the counter (OTC) antacids, OTC and prescription strength H_2_-receptor antagonists, prokinetic agents, and OTC and prescription strength proton pump inhibitors (PPI). The decision to initiate antireflux medications is typically driven by patient report of symptoms, and, in some cases, evidence of LPR-related changes (edema and erythema) to the mucosal tissue lining the surface of the larynx and pharynx, typically observed during laryngoendoscopic examination. Recently more and more studies are finding potentially negative effects of long term PPI usage [15, 16]. It is necessary then to determine if antireflux medications are warranted, necessary, and effective. Aside from symptom-driven diagnosis, additional objective data is needed in order to better understand the participant-specific manifestations of LPR [1, 5, 17–20].

Objective tests used for the diagnosis of gastroesophageal reflux disease (GERD) include barium swallow studies, esophagoscopy, esophageal motility testing, esophageal manometry, and pH monitoring [6]. Frye and Vaezi noted that upper gastrointestinal endoscopy and pH monitoring, when used to diagnose reflux in patients with symptoms not classic for GERD, have poor sensitivity and are not diagnostically helpful. They suggest an empiric trial of PPIs is a well-established, cost-effective tool [21]. In other expert opinion, Sataloff and colleagues [6] set forth prolonged pH monitoring as the most important method to quantify reflux and to determine whether a patient's symptoms are related to GERD or LPR. Oropharyngeal aerosol-detecting pH probe has been found to reliably document LPR events and was found to be better tolerated compared to the standard dual pH probe, which is traditionally positioned in the esophagus and may not be the best diagnostic tool for measuring the severity of LPR [22].

A pH of 4 has been used as a threshold in the distal esophageal pH monitoring [23]. There is a pH gradient in the esophagus when reflux occurs due to the neutralization of refluxed material by swallowed saliva. It is well known that the larynx is more susceptible to injury by lowered pH than the esophagus, as the larynx lacks both extrinsic and the intrinsic epithelial defenses of the esophagus [24]. The esophageal protective mechanisms include peristalsis, a mucosal structure that can better tolerate exposure to acid, and bicarbonate production, which helps prevent overacidity [6]. Therefore, the esophagus can tolerate a lower pH exposure than the larynx and upper airway.

Past investigations have attempted to establish abnormal pH thresholds for the pharynx and larynx [22, 23, 25, 26]. Ayazi and colleagues [23] found that the pattern of pharyngeal pH environment is significantly different in the upright and supine positions; therefore different thresholds are set based on body position. They also studied asymptomatic participants and analyzed pH at 0.5 intervals between 4 and 6.5 and found ranges for mild, moderate, and severe reflux during both upright and supine positioning [23]. This study found healthy group discriminatory pH thresholds were between 6.5 and 6.0 for mild upright reflux exposure, between 6.0 and 5.5 for moderate upright reflux exposure, and below 5.5 for severe upright reflux exposure. Likewise, the discriminatory pH thresholds were found to be between 6.0 and 5.5 for mild supine reflux exposure, between 5.5 and 5.0 for moderate reflux exposure, and below 5.0 for severe supine reflux exposure.

While the exposure of the mild and moderate pH levels in the upper airway may contribute to subtle tissue changes (e.g., posterior interarytenoid edema and erythema or accumulation of endolaryngeal mucous), the potential effects on voice quality, including hoarseness, loss of range, and vocal fatigue, are both highly variable and unpredictable. The performance demands placed on singers are considerable, requiring precise control of the larynx and upper respiratory structures, so even miniscule changes to vocal quality or endurance can be problematic [2–4, 8, 10, 27–33].

The goal of this study was to explore the relationship between subjective (Reflux Symptoms Index or RSI) and objective (oropharyngeal pH probe) measures of LPR severity in a cohort of professional and semiprofessional singers. We hypothesized that an inverse relationship existed between the RSI and pH probe testing results and that evaluated RSI scores would correspond to objective measures of lowered pH within defined ranges of mild, moderate, or severe LPR.

## 2. Methodology

This was a prospective, single-center study. Criteria for inclusion included men and women between 18 and 65 years of age that were singing professionally or semiprofessionally on a weekly basis, including college degree seeking vocal performance majors. Semiprofessionals were defined singers who use their singing voice professionally less than 10 hours per week and professionals were those who use their singing voice professionally more than 10 hours per week. All participants reported some degree of voice difficulty including, but not limited to, hoarseness, vocal fatigue, difficulty sustaining phonation while singing, and reductions in pitch range. All participants underwent videostroboscopic examination as part of their standard care, and other significant vocal pathologies were ruled out. Additionally, all participants were suspected, per the laryngologist, to have a possible cofactor of LPR based on either their RSI score and/or laryngeal imaging findings. Individuals were excluded from participation if they were under the age of 18 or over the age of 65, were hobby singers, had an organic vocal pathology, or were unable to wear the pH probe for at least 18 hours. All singers who were experiencing voice difficulty, without other major disease processes that could contribute to the symptoms, were included in the study. Antireflux medication use was not taken into consideration for this study as we sought to ascertain pH levels at the time of experiences the voice difficulty regardless of antireflux medication usage. All participants were recruited from The University of Central Florida's Voice Care Center in Orlando, FL, and the affiliated otolaryngology practice (Ear, Nose, Throat, and Plastic Surgery Associates' Voice Care Center). Informed consent from the University of Central Florida Institutional Review Board was obtained for each participant (IRB number: SBE-10-07001). Recruitment for this study was over a period of 6 months. This work was preliminarily based on the first author's master's thesis completed at the University of Central Florida in 2011 [34].

*Participant Perception.* The Reflux Symptom Index (RSI), a psychometrically validated 9-item questionnaire, was used to quantify participant's perceptions of laryngeal and pharyngeal reflux symptoms [19]. The RSI presents reflux related problems and asks participants to rate each problem along an ordinal scale, where 0 indicates “no problem” and 5 indicates a “severe problem.” Items from the RSI are presented as in Table 1. A raw score between 0 and 45 was generated by summing the responses for each of the nine variables. A score of 13 and above is considered to be abnormal.

*Oropharyngeal pH Measurement.* Immediately following completion of the RSI, each participant underwent an oropharyngeal pH monitoring study. The Dx-pH Measurement System™ (Respiratory Technology Corporation; ResTech) was used to directly measure liquid and gaseous pH levels in the oropharynx. An oropharyngeal probe was chosen for this study as it has been shown to correlate well with the gold-standard dual channel pH device that is placed through the pharynx and into the esophagus. The oropharyngeal probe has been said to be more comfortable compared to traditional pH monitoring, as the placement of the tip is in the upper oropharynx where awareness during swallowing is minimal and insertion does not require the patient to swallow the probe [26]. The following information was retrieved from the instructions for use for the Dx-pH Measurement System [35]. Prior to insertion, the sensor was calibrated in solutions with a pH of 7 and a pH of 4. This sensor was inserted into the nose and placed in the oropharynx behind the uvula. A lubricating gel was used to insert it into the nose for participant comfort. A light emitting diode (LED) flashed for the first two hours, which aided in the insertion and correct placement of the sensor. This technology includes dryout detection with hydration monitoring circuitry, which records a pH of 15 if a dryout period were to occur. The sensor was connected to a small microcomputer that was clipped to the waist, so that the participant could be monitored as they moved around in daily life. The participant presented to the clinical setting after 18–24 hours and the probe was removed. Extraesophageal placement of the pH probes within the pharynx, as opposed to the esophagus, distinguishes ResTech monitoring from other methods typically used in the diagnosis of GERD and therefore provides a more accurate, objective measure of LPR [25]. During testing voltage, potentials within the ResTech sensor change relative to the pH of aerosolized and liquid acids to which it is exposed [35]. Data, in the form of voltage readings, were recorded twice per second. Due to pH not remaining steady or reliable during meal times, the participants indicated eating times by pressing an assigned button on the device worn on the waist. These times were then excluded when analyzing the data. To account for postural changes that might affect probe readings, participants indicated when they entered a supine position for sleep by pressing a button on the ResTech device. Participants were encouraged to perform all daily activities as they normally would, as long as the activities did not interfere with the equipment. Specifically, they were encouraged to eat their regular diet and participate in their singing activities when they are able to. The thresholds and severity levels for normal and abnormal pH as outlined by Ayazi and colleagues were used when reporting this data and in the correlation in the current study.

All study personnel were blinded to the data. All data collected was deidentified. Participants were given a numeric code and the RSI and pH results were analyzed separately, prior to comparison and statistical analysis. All statistical analyses were completed using SPSS Version 19. A total of 13 response variables were extracted for analysis including total RSI score, individual items from the RSI (9 response variables), and pH monitoring (3 variables; duration of LPR episodes within mild, moderate, and severe pH ranges). Observed pH levels were subdelineated in to “mild,” (6.5–6.0 upright, 6.0–5.5 supine) “moderate” (6.0–5.5 upright, 5.5–5.0 supine), and “severe” (<5.5 upright, <5.0 supine) ranges, according to Ayazi and colleagues [23]. Data obtained during both upright and supine intervals were combined to generate a composite score for all participants.

Spearman correlation coefficient was used to determine if correlations existed between the pH severity and total RSI score, with further investigation with the separate variables of the RSI tool [36]. Spearman's correlation was used because the continuous variables in the pH data are not normally distributed and RSI are ordinal variables, which can be used with a nonparametric analysis, such as Spearman Rank Correlation Coefficient.

## 3. Results

Initially 21 participants were recruited for this study. One participant was excluded as he was unable to have the pH probe placed, due to a singing engagement. A total of 20 individuals (7 males, 13 females) completed all study procedures. Participants ranged in age from 18 to 62 (mean of 38.7 years, SD = 14). All reported singing either professionally (*n* = 13) or semiprofessionally (*n* = 7). Individual demographic data is presented in Table 2.

For participant perception (RSI), thirteen participants (65%) indicated a RSI raw score of 13 or above, which was determined to be abnormal, indicating the potential for the presence of reflux [17]. A breakdown of RSI response data is provided within Table 3.

ResTech pH measurement. Participants were monitored between 18 and 24 hours [mean of 22 hours, SD = 2]. No drying effect of the pH probe was recorded for any of the 20 participants and therefore the results accurately depict pH levels. Nineteen (95%) of participants demonstrated readings consistent with LPR during ResTech pH measurement. Of these, all demonstrated episodes of mild LPR. Episodes of moderate and severe LPR were demonstrated by fifteen (79%) and fourteen (74%) participants, respectively. A total of 3212 LPR episodes were recorded among all participants. Of these, 1946 (60.58%) were classified as “mild,” 785 (24.43%) “moderate,” and 481 (14.97%) “severe.” A total of 8765 minutes of LPR episodes were recorded among all participants. 3392 were classified as “mild” (38.69%), 1844 (21.03%) “moderate,” and 3529 (40.26%) “severe.” Descriptive data pertaining to LPR episodes is presented in Table 4.

As the the number of reflux episodes can be highly variable, lasting anywhere from less than one second to many hours, it was decided the focus of the correlation analysis would be on duration of reflux episodes at the various severity levels. No correlations were found between total RSI score and duration of reflux episodes. Result of the correlation analysis is listed in Table 5.

## 4. Discussion

This study sought to explore the relationship between subjective and objective measures of LPR severity in professional and semiprofessional singers. We hypothesized that significant effects existed between the RSI and pH probe testing results, and that RSI scores would exhibit significant positive correlations with objective measures of decreased pH within defined ranges of mild, moderate, or severe LPR. Although no well-established correlations existed between duration of reflux episodes and total RSI score, there is suggestion for a potential relationship between the presence of excessive mucous within the throat and duration (of moderate) LPR episodes (Spearman correlation coefficient *r*_*s*_ = 0.399, *p* = 0.041); however, a larger population is necessary to establish if a true relationship exists.

Sixty-five percent of participants had abnormal RSI scores (35% with a score that was within normal limits); however, 95% of participants had at least mild reflux findings on pH probe. This is a significant discrepancy and may suggest that the RSI instrument is not sensitive enough to pick up subtle symptoms that may occur in singers as a result of mild to moderate reflux that reaches the upper airway. Common voice complaints reported by professional and semiprofessional singers include vocal fatigue, loss of range, and difficulty phonating softly [37, 38]. As these symptoms are not part of the RSI questionnaire, more research using objective reflux measures and their relationship to singer specific questions is needed.

The amount of acceptable acid exposure or the acceptable amount of time for pH to be below certain thresholds is unknown and more research needed to determine how much acid exposure is normal and how much is abnormal. Two events per day of reflux below a pH of 4 have been reported in healthy controls [39–42], yet the total time of this exposure is unknown. In another study, 3 pharyngeal reflux events per week have been found to produce laryngeal damage, especially if a preexisting mucosal injury exists [43]. Again, time of these events is unknown. In the present study, the total number of reflux episodes at the 3 severity levels and the total time of those episodes was quite disjunct. As can be seen in Table 4, there were a total of 481 severe reflux episodes across all participants, which is 14.97% of all reflux episodes. However, when the duration of those episodes is examined, the total time of severe reflux exposure was 3529 minutes which is 40.26% of the time of lowered pH exposure across the 3 severity levels. This is a difference of 25.29%. This point warrants further investigation.

The Ryan score is a popular calculation done using the percentage of time of pharyngeal acid exposure below 5.5 in upright and 5.0 in supine position, as well as the number of episodes and the duration of the longest episode below these thresholds. It yields a standardized value and then compares that to the patient's calculated value. This analysis was not used in the present study due to the calculation only considering thresholds in the severe range and below. This is an important aspect to consider when using the Dx-pH Measurement System software, as mild and moderate pH levels are not taken into consideration with this analysis. Anecdotally, many medical practices, including the one associated with this study, defer to the manufacture's thresholds as a means by which a diagnosis is reached. We believe there is clinical value in considering mild and moderate pH levels, especially with singers who require pristine tissue health for the coordination of singing.

In this study, no relationship between hoarseness or voice “problem” (RSI item 1) and oropharyngeal pH levels was revealed. The absence of this finding, particularly among professional and semiprofessional singers, bears attention. Anecdotally, singers served in our clinics report enhanced awareness of subtle vocal changes. For a singer, hoarseness is not a subtle symptom, as this would be significantly deleterious to a singer's livelihood. In general, professional and semiprofessional singers may be more likely to seek medical attention for these subtle vocal disturbances sooner, rather than waiting for symptoms to worsen, potentially exacerbating the issue.

It is worth discussing that there were no correlations found between duration of lowered pH exposure and symptoms of heartburn, chest pain, indigestion, or stomach acid coming up (question 9 on the RSI). The threshold for severe pH exposure in the pharynx is 5.5 in upright and 5.0 in supine position [23] and the threshold for abnormal pH in the esophagus is <3.1 distal to <4 in the proximal [26]. Heartburn is typically associated with esophageal reflux and as the abnormal pH threshold for reflux increasing from the distal to the proximal esophagus and into the pharynx; it is not surprising that there were no correlations in this study. If there was a correlation one would expect to see a trend toward severe reflux in the pharynx in a population where more subtle reflux (between 5.5 and 7.0) was more predominately found.

There were also no correlations found between pH levels and RSI 7 “Troublesome or annoying cough” or RSI 2 “Clearing your throat.” Cough can not only be triggered by direct contact of the laryngeal mucosa with refluxate, as is the case with LPR, but GERD could cause indirect irritation to the larynx due to esophageal irritation caused by a vagal reflex [44]. This reflex can trigger a cough or throat clear, which in turn can cause mechanical trauma on the vocal folds resulting in mucosal irritation [6]. The etiology of cough can be highly variable, but if objective data shows signs of reflux in the pharynx, even mild reflux, this could warrant a work-up by gastroenterology to ascertain the health of the esophagus and potential contributing factors. This is especially true as pH is found to increase from the distal esophagus to the pharynx. Milder pH levels in the pharynx could yield more severe exposure in the esophagus, which could account for cough and other symptoms on the RSI. Again, a larger population would yield increased power to shed more light on this comparison.

Analysis of individual participants data produced some observations of note. Predominately mild LPR was observed with participant (9). However, this individual's RSI score was a 26, the highest of all the participants, suggestive of LPR. Likewise, participant (3) had an abnormal RSI total score of 21 but only had one mild reflux episode, lasting less than 1 minute. Participant (14) did not drop below a pH of 6.5 throughout the 22 hours and 35 minutes of the pH monitoring. Interestingly, this participant's RSI score was 22, which is in the abnormal range. Two explanations for this discrepancy include that the participant did not have reflux episodes during the time of the pH monitoring study but did shortly before the study, which influenced the values that they assigned to the RSI or perhaps the participant had other irritants to the larynx influencing the symptom severity on the RSI. In instances like this, alternate conditions such as phonotrauma or allergies may need to be considered as etiological factors relating to the individual's voice complaints.

Considering these false positive results is challenging. A few studies have looked at the specificity and sensitivity of the RSI tool in patients diagnosed with LPR by pharyngeal pH monitoring. Belafsky and colleagues studied a group of 25 patients experiencing voice difficulty who were diagnosed with LPR (confirmed by 24-hour double-probe pH monitoring, with proximal probe 1 cm above the UES) and 25 health controls. LPR patients were treated with BID PPIs for 6 months. They found that LPR patients initially had a significantly high RSI scores compared to controls. Following treatment, LPR patients' RSI scores approached that of the asymptomatic controls [19]. Mesallam and colleagues found significant differences between patients with voice difficulty and divided them into an LPR positive group and an LPR negative group, using the RSI and oropharyngeal pH monitoring (pH threshold was 5.0 supine and 5.5 upright) [24]. To date, no studies have specifically evaluated singers, specifically commented on false positive RSI score, or have used the RSI when considering higher thresholds of pH. More research is needed with singers, considering higher thresholds of pH. In the current study, LPR cannot be ruled out as a contributing factor with the participants who had a pH score below 7 and above 5.5. It is possible that they could have had reflux before the pH monitoring started, which may have influenced the RSI rating. This should also be carefully considered when formulating a plan of care. Participants (2), (3), (10), and (12) showed only mild pH exposure and, with the exception of participant (3), had RSI scores that were within normal limits. In cases like these perhaps diet and lifestyle modifications would suffice and medical management for LPR would not be necessary. In the case of participant (3) with an abnormal RSI score, perhaps testing for a longer period of time and correlating symptoms throughout the testing period would provide a better diagnostic picture of pH exposure and related symptoms. Regarding mild reflux exposure and the singer, although many singers have good singing technique, singers can overuse their voice, have poor speaking voice habits, or have poor vocal hygiene. This can cause irritation to the vocal folds and that coupled with mild exposure to reflux could be detrimental to a singer. It can be seen that participant (1) experienced lowered pH mostly in the mild range; they experienced moderate and severe reflux as well. Clinically this case may be treated differently than the mild refluxers previously mentioned, perhaps with diet and lifestyle modifications and medical management.

Participants (8) and (11) presented with a small number of severe reflux episodes; however, these episodes were of notably longer duration compared to all other participants. In contrast, participant (9) displayed a great number (145) of LPR episodes; however, these episodes occurred for very brief periods of time. Participant (4) had an interesting profile, with an RSI score of 12, which is considered by diagnostic standards as “within normal limits” or not suggestive of LPR [19]. Participant (4) experienced only 16 severe LPR episodes; however, those episodes were of exceptional long duration, lasting the majority of the length of the study, 1255 minutes (21 hours). Both number of and duration of LPR episodes at mild, moderate, and severe levels were considered in this study. However, only the duration of episodes was used in the correlational analysis, due to the high variability of the number of episodes and the duration of each episode as described above. These authors surmised that duration of time may be a more accurate representation of the severity of reflux exposure. At this time, the literature does not provide us with the minimum time of lowered pH, at any level, in the oropharynx that would be considered damaging. Research looking at time of lowered pH exposure at the various thresholds and quality of life measures, before and after treatment, this would likely provide insight into this question.

This work represents a pilot study and future work will include a control group of nonsingers as well as a larger sample size of singers of varying ages, genres of vocal performance, and levels of training. Future work will compare measures of LPR obtained during “active” (e.g., involved in daily rehearsal or performances) with “rest” (not actively performing or rehearsing) intervals. Other factors that may influence GER or LPR should be considered, including smoking, obesity, diet, and other lifestyle factors. The RSI tool requires the individual to respond to the following question: “Within the last MONTH, how did the following problems affect you?” Therefore, the participant's perceptions of reflux severity may not always coincide with the time that he or she underwent the pH monitoring study. Future studies should administer the RSI multiple times throughout the duration of ambulatory probe monitoring in order to better correlate changes in pH levels with measures of LPR symptom severity.

There were a number of limitations to the current study. Our study cohort was small, and we did not include a control group of nonsingers. A 24-hour oropharyngeal pH probe test is merely a small glimpse into the life of one that is being tested. Very small amounts of refluxed content can cause trauma and damage to the sensitive tissue of the larynx and pharynx [43]. Considering this, longer testing may be necessary to accurately diagnose and treat this disorder. In this study, examinations were between 18 and 24 hours; therefore, the number of reflux symptoms and total time of episodes could be skewed as a result of somewhat uneven duration of each pH procedure between the participants. Although clinically relevant and interesting, it is unknown what constitutes a significant amount of time of exposure of pH at thresholds <7 and >5.5. More research is needed in this area.

## 5. Conclusions

This work represents a preliminary effort to explore the relationship between subjective and objective measures of LPR severity in a cohort of singers. No relationships between symptoms on the RSI and exposure to varying pH levels in the oropharynx were observed. However, this work does highlight some interesting individual data findings. This study suggests that the individual items on the RSI may not be sensitive to the subtle changes in vocal abilities of singers. Development and validation of a new reflux scale may better serve this population and may yield more relationship to subtle evidence of reflux. The study showed that the ResTech pH probe was a useful tool to easily assess pH levels at different thresholds in the oropharynx. The acceptable amount of time for pH to be below certain thresholds is unknown and more research needs to be designed in order to determine how much acid exposure is normal and abnormal. There may be clinical value in considering mild and moderate pH levels in the oropharynx, especially with singers.

## Figures and Tables

**Table 1:** Reflux Severity Index. *Within the last month, how did the following problems affect you?*

(1) Hoarseness or a problem with your voice	0	1	2	3	4	5
(2) Clearing your throat	0	1	2	3	4	5
(3) Excess throat mucous	0	1	2	3	4	5
(4) Difficulty swallowing food, liquids, or pills	0	1	2	3	4	5
(5) Coughing after eating or after lying down	0	1	2	3	4	5
(6) Breathing difficulties or choking episodes	0	1	2	3	4	5
(7) Troublesome or annoying cough	0	1	2	3	4	5
(8) Sensations of something sticking in your throat or a lump in your throat	0	1	2	3	4	5
(9) Heartburn, chest pain, indigestion, or stomach acid coming up	0	1	2	3	4	5

Total score:						

**Table 2:** Individual demographic data.

Participant	Professional = P > 10 hours of professional singing per week; semiprofessional = S < 10 hours of professional singing per week	Age	Gender Male = M Female = F
(1)	P	21	F
(2)	P	18	F
(3)	P	22	F
(4)	P	30	F
(5)	P	44	M
(6)	S	55	F
(7)	P	52	M
(8)	P	39	F
(9)	P	32	M
(10)	S	29	M
(11)	P	27	M
(12)	P	31	F
(13)	S	23	F
(14)	S	62	F
(15)	P	41	M
(16)	S	45	F
(17)	P	59	F
(18)	S	34	M
(19)	S	58	F
(20)	P	52	F

	P = 13 S = 7	Mean 38.7	13 F 7 M

The table depicts the individual demographic data for each participant. Included is the level of performance, whether professional or semiprofessional, age, and gender.

**Table 3:** Reflux Symptom Index (RSI) descriptive data.

Participant	RSI 1	RSI 2	RSI 3	RSI 4	RSI 5	RSI 6	RSI 7	RSI 8	RSI 9	RSI total
(1)	1	3	1	4	2	0	0	0	2	13^*∗*^
(2)	3	1	1	0	0	0	1	4	1	11
(3)	4	3	1	3	1	4	0	1	4	21^*∗*^
(4)	3	2	3	0	0	0	1	0	3	12
(5)	4	2	2	0	2	1	1	2	0	14^*∗*^
(6)	5	3	3	1	3	1	1	0	2	19^*∗*^
(7)	4	4	3	0	2	0	1	1	3	18^*∗*^
(8)	4	1	0	0	0	0	0	0	0	5
(9)	4	4	3	3	3	2	4	2	1	26^*∗*^
(10)	2	3	3	0	0	0	0	0	4	12
(11)	3	5	5	2	1	2	0	3	2	23^*∗*^
(12)	0	0	1	0	0	0	0	0	0	1
(13)	4	3	3	1	0	0	0	1	2	14^*∗*^
(14)	4	4	4	1	3	2	0	4	0	22^*∗*^
(15)	1	2	2	0	0	0	0	0	0	5
(16)	4	3	3	0	1	1	0	0	3	15^*∗*^
(17)	5	5	4	3	3	3	2	4	4	33^*∗*^
(18)	4	3	5	1	0	0	0	3	1	17^*∗*^
(19)	5	4	3	2	4	4	5	4	0	31^*∗*^
(20)	3	2	0	0	0	0	0	0	1	6

Mean	3.35	2.85	2.5	1.05	1.25	1	0.8	1.45	1.65	15.9
SD	1.38	1.30	1.46	1.31	1.37	1.37	1.39	1.63	1.46	8.52
Range	0–5	0–5	0–5	0–4	0–4	0–4	0–5	0–4	0–4	1–33

The table depicts the descriptive data for all participants for the RSI including individual scores for each variable, total RSI score for each participant, mean and standard deviation for all variables, and range of scores for all participants. The values that are asterisked and bolded depict an abnormal score on the RSI.

**Table 4:** Individual data for pH results.

Participant	Time total (min)	Time mild	Time mod	Time sev	# mild	# mod	# sev
(1)	1404	344	38.1	2.1	238	32	7
(2)	1417	7.1	0	0	8	0	0
(3)	1149	0.1	0	0	2	0	0
(4)	1306	5.1	46.5	1255.2	1	5	16
(5)	1363	22.6	20.9	13.4	25	4	2
(6)	1375	293.8	207.9	218.9	267	174	104
(7)	1335	220	141.1	109.1	26	49	19
(8)	1125	55.3	2.8	119.6	58	4	6
(9)	1141	76.7	0.8	0	146	2	0
(10)	1378	13.2	0	0	61	0	0
(11)	1128	272	396.3	572	108	13	83
(12)	1348	0.2	0	0	4	0	0
(13)	1425	75.8	70	270	62	24	10
(14)	1341	0	0	0	0	0	0
(15)	1395	402.9	81.3	60.8	117	66	2
(16)	1435	211.5	278.9	413.8	154	66	152
(17)	1435	37.1	0.8	0.5	261	2	3
(18)	1379	481.3	323.8	353	136	215	43
(19)	1370	603.2	167.1	130	212	89	30
(20)	1380	270.1	67.7	10.6	60	40	4

Total	26629	3392	1844	3529	1946	785	481
Mean	1331	92.2	176.45	97.3	39.25	24.05	169.6
SD	108	121.58	302.94	90.74	59.89	41.56	184.11
Range	1125–1435	0–603.20	0–396.30	0–1255.20	0–267.00	0–215.00	0–152.00

The table depicts the total duration (in minutes) of each pharyngeal pH monitoring study, duration, and number of mild, moderate, and severe LPR episodes as well as the range of duration and time.

**Table 5:** Spearman correlation coefficient, *N* = 20.

	Duration of mild reflux episodes	Duration of moderate reflux episodes	Duration of severe reflux episodes
RSI total	*r* = 0.156 *p* = 0.509	*r* = 0.209 *p* = 0.375	*r* = 0.042 *p* = 0.859
RSI 1	*r* = 0.134 *p* = 0.287	*r* = 0.237 *p* = 0.158	*r* = 0.214 *p* = 0.182
RSI 2	*r* = 0.252 *p* = 0.142	*r* = 0.276 *p* = 0.120	*r* = 0.090 *p* = 0.353
RSI 3	*r* = 0.170 *p* = 0.236	**r** = 0.399^*∗*^ **p** = 0.041	*r* = 0.363 *p* = 0.058
RSI 4	*r* = 0.212 *p* = 0.185	*r* = 0.031 *p* = 0.448	*r* = 0.112 *p* = 0.319
RSI 5	*r* = 0.162 *p* = 0.247	*r* = 0.079 *p* = 0.370	*r* = −0.112 *p* = 0.319
RSI 6	*r* = −0.061 *p* = 0.399	*r* = −0.012 *p* = 0.479	*r* = −0.107 *p* = 0.327
RSI 7	*r* = 0.072 *p* = 0.381	*r* = −0.001 *p* = 0.499	*r* = −0.022 *p* = 0.464
RSI 8	*r* = −0.057 *p* = 0.405	*r* = −0.069 *p* = 0.387	*r* = −0.153 *p* = 0.260
RSI 9	*r* = −0.143 *p* = 0.274	*r* = 0.025 *p* = 0.458	*r* = 0.092 *p* = 0.350

The table depicts Spearman correlation coefficient results for RSI total and total time of reflux in the mild, moderate, and severe severity pH levels. This table also shows results of the individual variables on the RSI with total time at the 3 severity levels. The asterisk and bolding indicates a potentially statistically significant result.
